# Comparing Actual and Rounded Serum Creatinine Concentration for Assessing the Accuracy of Vancomycin Dosing in Elderly Patients: A Single-Center Retrospective Study

**DOI:** 10.3390/healthcare12111144

**Published:** 2024-06-04

**Authors:** Rawan Bukhari, Hani Hasan, Doaa Aljefri, Rawan Rambo, Ghusun AlSenaini, Yahya A. Alzahrani, Abdullah M. Alzahrani

**Affiliations:** 1 Pharmaceutical Care Department, Ministry of National Guard—Health Affairs, Jeddah 22384, Saudi Arabia; bukharira2@mngha.med.sa (R.B.); hasanhi@mngha.med.sa (H.H.); rambora01@mngha.med.sa (R.R.); alsenainigh@mngha.med.sa (G.A.); 2 King Abdullah International Medical Research Center, Jeddah 21423, Saudi Arabia; 3 College of Medicine, King Saud bin Abdulaziz University for Health Sciences, Jeddah 22384, Saudi Arabia; 4 Pharmaceutical Care Division, King Faisal Specialist Hospital & Research Centre, Jeddah 23433, Saudi Arabia; daljefri@kfshrc.edu.sa; 5 Drug Information Center, Department of Pharmacy, East Jeddah Hospital, Ministry of Health, Jeddah 22253, Saudi Arabia; yalzahrani5@moh.gov.sa

**Keywords:** estimated creatinine clearance, vancomycin, elderly, kidney function, rounded serum creatinine, actual serum creatinine

## Abstract

Prescribers often face the challenge of predicting creatinine clearance (CrCl) in elderly patients who are 65 years or older and have serum creatinine (SCr) concentrations below 1 mg/dL. Studies have shown that utilizing rounded SCr would underestimate CrCl in this population, which could lead to the under-dosing of some medications like vancomycin. The current study aimed to compare the accuracy of vancomycin dosing using actual SCr versus rounded SCr to 1 mg/dL in elderly patients. A total of 245 patients were included. The therapeutic trough level (10–20 mg/L) was achieved in 138 (56.3%) patients using actual SCr. Sub-therapeutic (<10 mg/L) and supra-therapeutic (>20 mg/L) trough levels were observed in 32 (13.1%) and 75 (30.6%) patients, respectively. The predictive performance of different vancomycin doses based on actual SCr and rounded SCr compared to the targeted maintenance dose (TMD) showed a stronger correlation of dosing based on actual SCr with TMD (r = 0.55 vs. 0.31) compared to rounded SCr dosing; both doses showed similar precision, with ranges of ±552 mg/day for the dosing based on actual SCr and ±691 mg/day for the dosing based on rounded SCr. Furthermore, the dosing based on actual SCr showed a lower error percentage (69%) and a higher accuracy rate (57.6%) within ±10% of the TMD compared to the dosing based on rounded SCr, which had an error percentage of (92.3%) and an accuracy rate of (40%). The prevalence of vancomycin-associated nephrotoxicity (VAN) was seen in 44 (18%) patients. Patients between 75 and 84 years of age, those who were bedridden, and those with vancomycin trough concentrations greater than 20 mg/L had a higher risk of developing VAN. In conclusion, in elderly patients, estimating vancomycin dosing based on actual SCr was more accurate compared to rounded SCr to 1 mg/dL. The efficacy of vancomycin could be negatively affected by rounding up SCr, which could underestimate CrCl and result in the under-dosing of vancomycin.

## 1. Introduction

An appropriate assessment of kidney function in elderly patients is crucial to determine the appropriate dosage of medications and predict the likelihood of developing acute kidney injury (AKI) [1]. Prescribers often face the challenge of predicting creatinine clearance (CrCl) in elderly patients who are 65 years or older and have a serum creatinine (SCr) concentration below 1 mg/dL [2]. The Cockcroft–Gault (CG) equation was initially formulated in 1976. It utilizes patient-specific variables including age, gender, weight, and steady-state serum creatinine (SCr) concentrations to estimate creatinine clearance (CrCl) over a 24 h period. The derivation of this equation was based on a cohort of 249 male Caucasian patients. Notably, only 23.7% of the participants were over the age of 70 years, representing a significant limitation of this broadly applied pharmacokinetic equation [3]. In general, elderly patients with poor nutritional status and reduced muscle mass produce less creatinine, resulting in lower SCr concentration; SCr is a critical factor in this equation [4]. With low SCr in the elderly, the use of CG equations can overestimate renal function in this population [5]. Finney et al. discovered that the CG equation does not accurately assess kidney function in older people [6].

In many institutions, prescribers have a standard practice of rounding SCr concentration to 1 mg/dL if SCr is below 1 in elderly patients [7,8]. This practice is applied to some antibiotics such as vancomycin, aminoglycosides, and beta-lactam antibiotics. Results from an earlier study demonstrated that utilizing the rounded SCr to 1 mg/dL would significantly underestimate CrCl and decrease the antibiotic dosing that requires renal adjustments [9].

The pharmacokinetic profile of vancomycin in elderly patients differs significantly from that of younger patients [10,11]. Elderly patients have slower clearance and a smaller volume of distribution. They also have decreased albumin levels, which could lead to an increase in the incidence of nephrotoxicity [11].

Few published studies have been conducted to address the impact of utilizing rounded SCr practice on vancomycin dosing in elderly patients. Young et al. compared the measured vancomycin trough level at steady state with the predicted trough level based on rounded versus actual SCr [5]. While the study by Young et al. provided valuable insights into the implications of SCr rounding on the prediction of vancomycin trough levels, revealing that actual SCr values may enhance dosing accuracy, it was constrained by its modest sample size and a selection of patients that might not have captured the full spectrum of the elderly demographic. Additionally, the Young et al. study did not explore in depth the risk factors of nephrotoxicity, resulting in a notable gap in understanding where patients could be more susceptible to renal complications under vancomycin treatment.

The current study aimed to evaluate the impact of using rounded SCr values (rounded up to 1 mg/dL) versus actual values on the accuracy of initial vancomycin dosing achieving the target maintenance dose in elderly patients aged 65 and above. The risk factors for vancomycin-associated nephrotoxicity (VAN) were also investigated.

## 2. Materials and Methods

### 2.1. Study Design, Place, and Setting

This was a retrospective study carried out at King Abdulaziz Medical City, Jeddah (KAMC-J), in the inpatient setting between 1 January 2019 and December 2022. The proposal was approved by the institutional review board (IRB) at King Abdullah International Medical Research Center (KAIMRC) (IRB/1688/22). Demographic and clinical data for this study were sourced from the Hospital Information System (HIS) and collected on a Microsoft Office Excel 2010 sheet protected by a password key to ensure the confidentiality of data, encompassing variables like gender, age (divided into three subgroups), body weight, height, albumin levels, and serum creatinine (SCr). The dataset also included measured vancomycin trough concentrations, target maintenance doses (TMDs), and total daily doses (TDDs) based on actual SCr values. Furthermore, body mass index (BMI) and baseline creatinine clearance (CrCl) were calculated as described in Section 2.3.

### 2.2. Criteria of Inclusion and Exclusion

All elderly patients aged 65 years or older who received intravenous vancomycin empirically or therapeutically, with normal renal function defined as an eGFR greater than or equal to 90 mL/min/1.73 m² and serum creatinine (SCr) less than 1 mg/dL [12], admitted under the medical or surgical unit, with initial vancomycin trough levels measured at steady state collected prior to the third or fourth dose were included. This included patients with initial trough levels below 10 mg/L, within 10–20 mg/L, and above 20 mg/L to capture real-world clinical variations and subsequent dose adjustments.

All elderly patients who were admitted to other units, discontinued vancomycin before achieving therapeutic trough levels, who had unstable renal function, and who had muscular dystrophy, spinal cord injury, and amputations were excluded.

### 2.3. Study Equations and Definitions

The CrCl was calculated using the Cockcroft–Gault (CG) equation: CrCl (mL/min) = [(140 − age) × weight (kg)]/(72 × serum creatinine [SCr] in mg/dL) and, for females, this result was multiplied by 0.85. In non-obese patients, the ideal body weight (IBW) was utilized to calculate CrCl. When a patient’s total body weight (TBW) was less than their IBW, TBW was used instead [8]. For obese patients (BMI greater than 30 kg/m^2^), the adjusted body weight (ABW) was applied to the equation as per the guidelines [8]. BMI was calculated by taking the patient’s weight, in kilograms, divided by their height, in meters squared (BMI = weight (in kg)/height^2^ (in m)) [13].

The therapeutic vancomycin trough level was defined as a level between 10 and 20 mg/L at a steady state, in accordance with institutional guidelines (Supplementary Materials). Levels below 10 mg/L were considered sub-therapeutic, while levels exceeding 20 mg/L were deemed supra-therapeutic. Vancomycin doses were determined in clinical practice by using the patient’s CrCl based on actual SCr and multiplying it by their TBW, rounding to the nearest 250 mg [14]. The dosing interval was determined according to the patient’s CrCl based on actual SCr, following institutional guidelines (see Supplementary Materials). The target maintenance dose (TMD) of vancomycin was the amount needed to achieve a therapeutic trough level (10–20 mg/L) at a steady state. This dose was fine-tuned through various adjustments in response to measured trough levels until the therapeutic level was reached. Vancomycin dose and interval, determined by CrCl calculated using rounded SCr values (rounding SCr values less than 1 mg/dL up to 1 mg/dL), were then obtained for further analysis and comparison [5].

In the present work, renal function instability was defined as the presence of chronic kidney disease (CKD), end-stage renal disease (ESRD), or acute kidney injury (AKI) or a documented change in SCr of 25% or more before a vancomycin dose was given.

All definitions for this study are provided in the Supplementary Materials for easy reference.

### 2.4. Endpoints

#### 2.4.1. Primary Endpoints

- To determine the percentage of patients who achieved therapeutic vancomycin trough levels using the actual SCr.
- To predict the performance of vancomycin doses based on actual and rounded SCr to TMD using the following measuring tools: correlation, bias, precision, error, and accuracy.

#### 2.4.2. Secondary Endpoints

- To evaluate the incidence of vancomycin-associated nephrotoxicity (VAN).
- To evaluate the risk factors of VAN.

### 2.5. Statistical Analysis

All statistical computations were performed using Statistical Package for the Social Sciences (SPSS) version 26.0 (SPSS Inc., Chicago, IL, USA). Categorical variables were expressed using frequencies and percentages, while the mean ± (standard deviation) or median (interquartile range) was used to present continuous variables. The mean values of vancomycin dose, coefficient of variation, correlation, bias, precision, error percentage, and accuracy were calculated for both actual and rounded doses. Additionally, a chi-square test was performed to examine the significant difference in accuracy between the two groups [15]. Odds ratios (ORs) with 95% confidence intervals (CIs) were calculated to determine the increased odds of accuracy between the actual and rounded dose groups. Multifactor logistic regression analysis was performed to identify predictors of the risk factors for VAN. Odds ratios were calculated to determine the association between these variables and VAN. All reported *p*-values were two-sided and a *p*-value of <0.05 was considered statistically significant.

## 3. Results

### 3.1. Patient Characteristics

**Figure 1:**
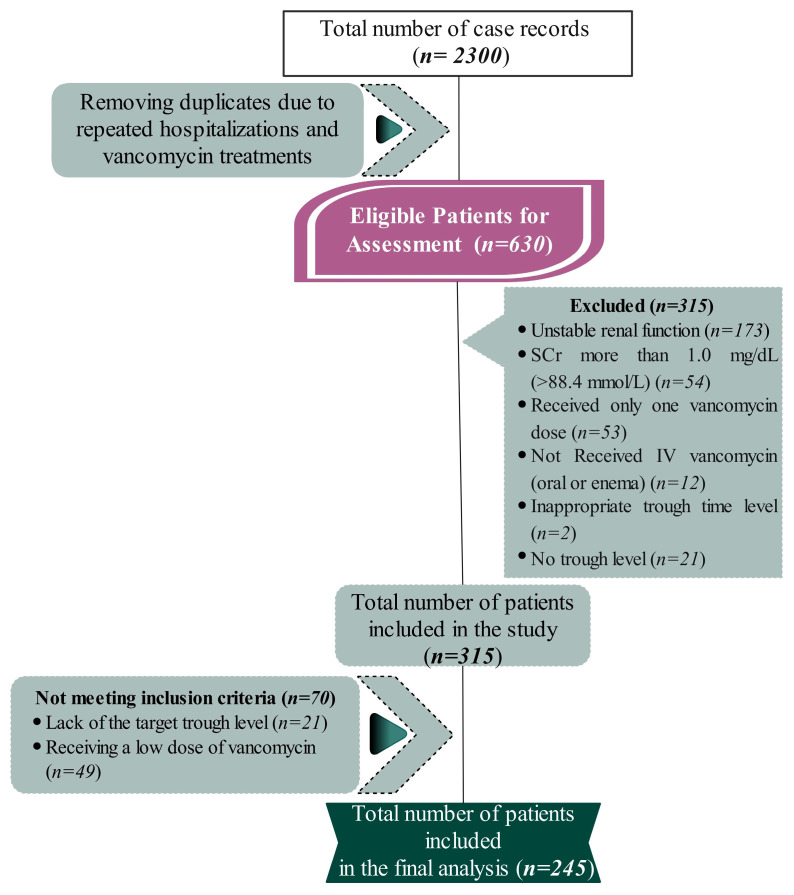
The flowchart illustrates the process of participant selection, beginning with an initial pool of 2300 case records. After removing duplicates due to repeated hospitalizations and vancomycin treatments, 630 patients were evaluated for eligibility according to the predefined criteria. A total of 385 participants were excluded based on the exclusion criteria, including unstable renal function and serum creatinine levels greater than 1.0 mg/dL. The final eligible patients were refined to 245 participants for the final analysis of the study.

Initially, 2300 case records were collected. After removing duplicates due to repeated hospitalizations and vancomycin treatments, 630 patients were evaluated for eligibility according to the predefined criteria. Of these, 315 (50%) satisfied the inclusion criteria. The most common reasons for exclusion were patients with unstable renal function (*n* = 173), those receiving only one dose of vancomycin (*n* = 53), and those with SCr of more than 1 mg/dL (*n* = 54). Of the 315 included patients, 70 (22.2%) were excluded for not achieving the therapeutic trough level (*n* = 21) and receiving a suboptimal starting dose of less than 15 mg/kg/dose of vancomycin (*n* = 49). A total of 245 patients were included in the final analysis (Figure 1).

Patient demographics and clinical characteristics are summarized in Table 1. The mean age of included patients was 78.71 (±9.09) years old and the majority of patients were male (77%). The median baseline SCr and corresponding CrCl were 0.72 mg/dL and 65 mL/min, respectively. The mean initial dose of vancomycin was 1695 mg ± 583 and the median trough level was 16.6 mg/L (12.3–21.5).

### 3.2. Percentage of Patients Who Achieved Therapeutic Vancomycin trough Levels Based on Actual SCr

**Figure 2:**
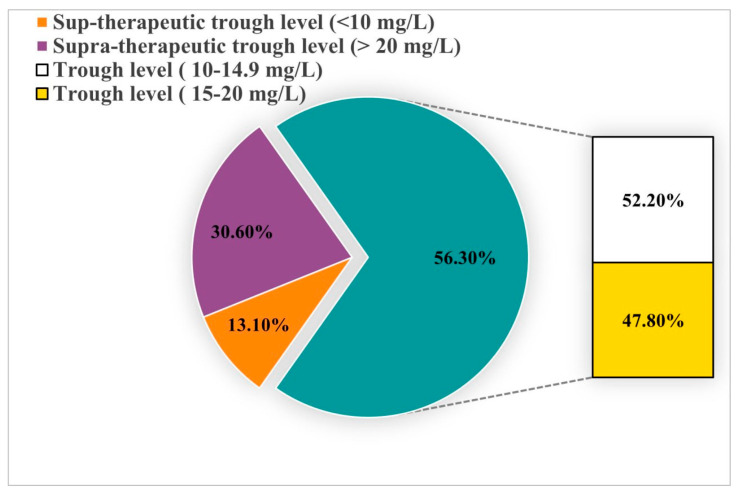
This pie chart displays the distribution of vancomycin trough levels in elderly patients, categorizing them into sub-therapeutic (<10 mg/L), supra-therapeutic (>20 mg/L), and therapeutic ranges (10–14.9 mg/L and 15–20 mg/L).

The therapeutic trough level (10–20 mg/L) was documented in 138 (56.3%) patients. Out of 138 patients, 72 (52.2%) had a trough level between 10 and 14.9 mg/L, while 66 (47.8%) patients had it between 15 and 20 mg/L. Subtherapeutic and supratherapeutic trough levels were seen in 32 (13.1%) and 75 (30.6%) patients, respectively (Figure 2).

### 3.3. Predictive Performance of Vancomycin Dosing Based on Actual and Rounded SCr Compared to TMD

Vancomycin total daily dose (TDD) based on actual SCr had a mean value of 1695 mg/day ± 583, while TDD based on rounded SCr had a lower mean value of 1487 mg/day ± 702. The correlation coefficient was moderate between the TMD and dosing based on actual SCr (r = 0.55), but weak with dosing based on rounded SCr (r = 0.31). The bias was negative for the actual SCr dose, with a difference of 190 mg/day, and positive for the rounded SCr dose, with a difference of 17.4 mg/day. The precision was similar for dosing based on both actual and rounded SCr, with a range of ±552 and ±691 mg/day, respectively. The error percentage was 69% for the actual SCr dose and 92.3% for the rounded SCr dose. The accuracy of dosing based on actual and rounded SCr within ±10% and ±15% of the TMD was 57.6%, 58.4%, 40%, and 40.4%, respectively. However, the dose based on actual SCr had a higher accuracy rate of 65.7% within ±30% of the TMD, while the dose based on rounded SCr had a rate of 56.7% (Table 2).

The result of χ ^2^ showed a significant difference between the two groups in the accuracy rate at 10%, 15%, and 30% of χ ^2^ (1, N = 245) = 12.8, χ ^2^ (1, N = 245) = 14.1, and χ ^2^ (1, N = 245) = 10.03 (*p* < 0.05), respectively. Furthermore, there was an increased odds of the accuracy among doses based on actual SCr at 10%, 15%, and 30% (OR = 2.6, CI 95% = 1.5–4.6; OR = 2.8, CI 95% = 1.6–4.8; and OR = 2.3, CI 95% = 1.4–4.1 (*p* < 0.05), respectively) compared to a dose based on rounded SCr.

### 3.4. Incidence and Risk Factors of Vancomycin-Associated Nephrotoxicity (VAN)

VAN was observed in 44 (18%) patients. Table 3 demonstrates the characteristics of patients who developed VAN. The mean age of the VAN group was 81 years, compared to 78 years in the non-VAN group. BMI was slightly lower in the VAN group (25.3 ± 5.8 vs. 26.3 ± 7.6). The mean SCr was higher in the VAN group (0.72 mg/dL ± 0.13 vs. 0.69 mg/dL ± 0.12). The mean vancomycin trough level was slightly higher in the VAN group (18.9 mg/L ± 7.3 vs. 17 mg/L ± 7.5). Lower albumin levels were also found in the VAN group (26.9 g/L ± 5.8 vs. 28.14 g/L ± 6.2).

**Figure 3:**
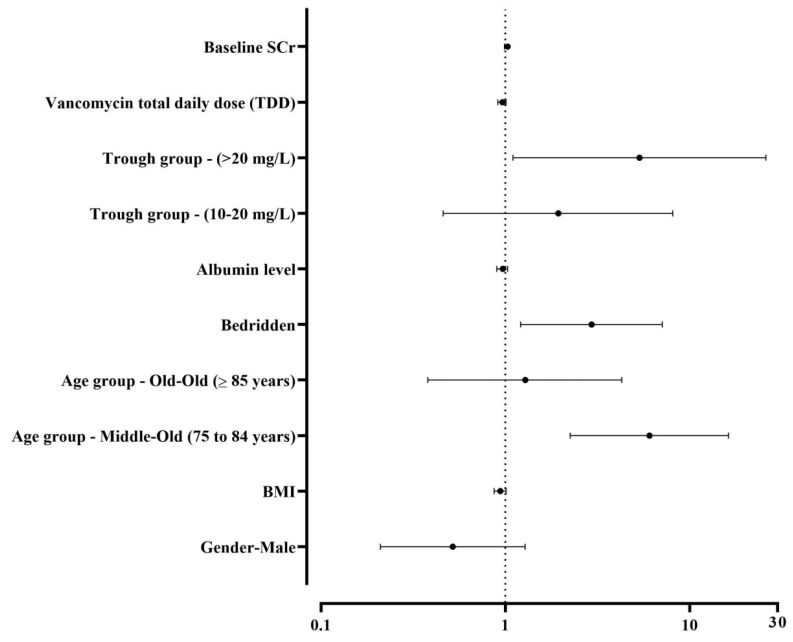
Forest plot of the odds ratios (ORs) for various predictors of nephrotoxicity in patients treated with vancomycin. This plot includes baseline serum creatinine (SCr), total daily dose (TDD) of vancomycin, trough concentration groups, albumin levels, mobility status (bedridden), age categories, body mass index (BMI), and gender. The vertical dotted line at OR = 1 indicates no effect. Values to the right of this line suggest an increased risk, while values to the left suggest a decreased risk. Confidence intervals that cross this line imply statistical non-significance. The trough concentration group <10 mg/L was used as the reference for comparison, while the age group <75 years was the reference group for comparison.

A multifactor logistic regression analysis was performed to identify predictors of the risk factors for VAN. The predictor variables included gender, albumin level, baseline SCr, loading dose administration, bedridden status, vancomycin TDD, CrCl at baseline, age groups (young, middle, and old), BMI, and trough levels. The results showed that three variables were statistically significant predictors of VAN with a *p*-value of 0.05: patients between 75 and 84 years old, bedridden patients, and those who had troughs higher than 20 mg/L were at a higher risk of VAN (Figure 3).

## 4. Discussion

Predicting renal function in the elderly is still uncertain. The current study found that the therapeutic trough level was achieved in 138 patients using vancomycin dosing based on actual SCr, representing 56.3% of the total. The current work compared vancomycin dosing based on actual and rounded SCr. The results showed that both methods had similar levels of precision. The dose based on actual SCr had a range of ±552 mg/day, while the dose based on rounded SCr had a range of ±691 mg/day. In addition, the dosing based on actual SCr had a lower error percentage (69% vs. 92.3%) and higher accuracy rate (65.7% vs. 56.7%) within ±30% of the TMD compared to the dosing based on rounded SCr. Furthermore, the prevalence of VAN in this study was 44 patients (18%) and the logistic regression model was used to analyze predictors of VAN; the results showed that patients between 75 and 84 years old, bedridden patients, and those who had troughs higher than 20 mg/L were at a higher risk of VAN.

According to this study’s findings, 56.3% of patients who received a vancomycin dose based on their actual SCr reached the therapeutic trough level (10–20 mg/L). This indicates that only half of the patients were able to reach the desired range. Young et al. evaluated the difference between predicted trough levels based on rounded SCr and actual SCr. The majority of patients, representing (92.9%), who used rounded SCr had subtherapeutic vancomycin troughs [5]. However, the study had a small sample size and aimed for a target trough level of 15–20 mg/L. They concluded that the measured trough was more accurately predicted by using actual SCr compared to SCr rounded to 1 mg/dL in elderly patients with SCr below 1 mg/dL [5].

In the present study, however, 30.6% of patients had trough levels above 20 mg/L, indicating potential overdosing or slowed drug metabolism. This raises concerns about the safety of the current dosage and monitoring methods. Studies have shown that some elderly patients have a longer half-life for vancomycin compared to younger individuals. Furthermore, it has been found that older people have a higher volume of distribution and lower clearance, which can lead to the accumulation of vancomycin and higher trough levels [16,17].

The current work evaluated the performance of vancomycin dosing based on actual SCr versus rounded SCr compared to the TMD based on several measuring tools. Both methods showed similar levels of precision. However, the dosing based on actual SCr had a lower error percentage (69% vs. 92.3%) and had markedly higher accuracy rates within ±10%, ±15%, and ±30% of the target maintenance dose (57.6%, 58.4%, 65.7%) than dosing based on rounded SCr (40%, 40.4%, 56.7%). When comparing SCr rounded to 1 mg/dL, actual SCr was a more accurate indicator of a patient’s renal function. Additionally, dosing based on actual SCr showed a moderate correlation with TMD (r = 0.55) compared to rounding SCr dosing (r = 0.31). These results are consistent with those of Bertino et al., where a greater portion of the variance of CrCl was explained by the use of measured SCr (r^2^ = 0.68 vs. r^2^ = 0.32) versus the use of SCr rounded to 85 μmol/L (0.9 mg/dL). They concluded that when estimating CrCl using the CG equation in patients with SCr of 85 mmol/L (0.9 mg/dL), the actual SCr should be utilized [18]. In addition, this study supports Nguyen et al.’s recommendation to utilize actual Scr rather than rounding it to determine renal function in older people since rounding SCr results in inaccurate assessments of kidney function and drug dosing problems [19].

Clinicians must strike a compromise between pharmacodynamic goals and the risk of nephrotoxicity while administering vancomycin. In the present study, the prevalence of VAN was found to be 18%, which is consistent with the range of 5% to 43% reported in meta-analysis studies [20]. The variation in prevalence across studies may be due to differences in patient populations, study designs, and definitions of nephrotoxicity [21]. Similarly, a retrospective investigation comparing the risk of VAN in elderly versus younger patients found that elderly individuals had a higher rate of nephrotoxicity than younger patients, occurring in 7.8% vs. 18.9% of patients; this difference was thought to be due to an unequal distribution of other risk factors, like the use of loop diuretics [21]. However, this difference was greater than in other similar studies. Young et al. found that nephrotoxicity was observed in 7.1% of patients, but this could have been owing to the limited sample size [5].

The predictors of VAN in the current study were being bedridden, the middle-old age group (75 to 84 years old), and trough levels higher than 20 mg/L. A recent study by Wang Y. et al. found that nearly 12.0% of elderly patients experienced nephrotoxicity after being treated with vancomycin. The factors independently linked to vancomycin trough levels ≥ 15 mg/L, treatment duration of 15 days or more, and co-administration of aminoglycosides [17].

One explanation for the bedridden in the current study is that prolonged bed rest facilitates a reduction in protein synthesis and an accelerated loss of muscle mass [22]. Reduced muscle mass can affect laboratory values such as SCr, which, in turn, impacts common calculations used to estimate CrCl (such as the CG equation) [23]. These findings suggest that for frail patients with low muscle mass, the prescriber may need to adjust for when very low SCr concentrations (<0.5 mg/dL) are measured by, for example, rounding the value to 0.68 mg/dL, in order to calculate CrCl more accurately. Such adjustments could help account for the impact of low muscle mass on creatinine concentration and produce a more precise estimate of kidney function in this population of patients [24].

## 5. Conclusions

In conclusion, this study provides information on the effects of serum creatinine rounding in elderly populations. While previous studies examined SCr rounding in a smaller cohort, our research expands the understanding of this issue to a broader demographic that is more representative of elderly patients aged 65 years and older. Using actual SCr measurements rather than values rounded to 1 mg/dL enabled a more accurate initial vancomycin dose to be given to achieve target trough concentrations. Furthermore, this study investigated risk factors that can predispose patients to VAN. The results suggest that rounding SCr values can underestimate renal function in older adults and lead to the under-dosing of vancomycin, which may negatively impact efficacy. Therefore, the use of actual SCr values could lead to more optimized vancomycin doses in elderly patients, and the monitoring of risk factors for nephrotoxicity allows for safer administration and better patient outcomes.

## 6. Limitation

This study provided valuable insights into the dosing of vancomycin in elderly patients, yet it had several limitations that were considered when interpreting the results. The retrospective nature inherently limited our ability to control for all potential confounding variables, and data collection from a single tertiary care center might not reflect practices or patient populations at other institutions, thereby limiting generalizability. The selection criteria and the specific setting may have introduced selection bias, as patients excluded due to incomplete data or those not meeting the inclusion criteria could have differed significantly from those included. The practice of rounding serum creatinine to 1 mg/dL, while clinically justified, introduced a methodological bias by assuming a uniform impact on renal function estimation across all patients, which might not have accurately reflected individual variations. Additionally, the absence of long-term follow-up data prevented us from assessing the sustained impacts of the dosing strategy, particularly concerning renal function recovery or long-term nephrotoxicity. Finally, other variables such as concurrent medications, nutritional status, and hydration levels, which can significantly affect vancomycin pharmacokinetics and nephrotoxicity, were not fully accounted for in our analysis. Future research should consider these aspects to enhance the precision of dosing recommendations and further our understanding of VAN in elderly patients.

## Figures and Tables

**Table 1:** Descriptive table of demographic variables and clinical characteristics.

Variable	*n* (%), Mean ± SD, or Median [Q1, Q3]
Male	189 (77)
Female	56 (23)
Age (years)	78.71 ± 9.09
Young-Old (65 to 74 years) *n* (%)	92 (37.6)
Middle-Old (75 to 84 years) *n* (%)	92 (37.6)
Old-Old (≥85 years) *n* (%)	61 (24.8)
TMD of vancomycin (mg)	1505 ± 579
TDD of vancomycin based on actual SCr (mg)	1695 ± 583
TDD of vancomycin based on round SCr (mg)	1487 ± 702
Height (cm)	162.3 ± 10.7
	**Median [Q1, Q3]**
Body weight (kg)	65 (55–73.9)
BMI (kg/m^2^)	24.6 (21–29.6)
Albumin (g/L)	28 (24–31)
SCr (mg/dL)	0.72 (0.62–0.84)
CrCl at baseline (mL/min)	65 (54–83.5)
CrCl using rounded SCr (mL/min)	48 (40–56)
Measured vancomycin trough (mg/L)	16.6 (12.3–21.5)

This table summarizes the demographic and clinical characteristics of the study participants. Data are presented as number (percentage), mean ± standard deviation (SD), or median and interquartile ranges (Q1, Q3), as appropriate, illustrating the demographic and clinical parameters of the participants involved in this study. BMI: body mass index; SCr: serum creatinine; CrCl: creatinine clearance; TMD: target maintenance dose; TDD: total daily dose.

**Table 2:** Predictive performance of vancomycin dosing based on actual and rounded SCr compared to TMD.

Indicators	TMD	Dosing Based on Actual SCr	Dosing Based on Rounded SCr
Vancomycin dose (mg/day)	1505 ± 579	1695 ± 583	1487 ± 702
Coefficient of variation (%)	38.4	34.3	47.2
Correlation		0.55	0.43
Bias (mg/day)		−190	17.4
Precision (mg/day)		±552	±691
Error (%)		69	92.3
Accuracy			
±10%		57.6	40
±15%		58.4	40.4
±30%		65.7	56.7

This table presents a comparative analysis of vancomycin dosing strategies, evaluating metrics such as dose, variability, correlation, bias, precision, error, and accuracy when dosing was based on actual or rounded serum creatinine levels (SCr). SCr: serum creatinine; TMD: target maintenance dose.

**Table 3:** Characteristics of patients with/without vancomycin-associated nephrotoxicity.

Variable	Non-VAN (*n* = 201)	VAN (*n* = 44)	*p*-Value
Male, *n* (%)	135 (67.2)	30 (68.2)	0.8
Age, years (mean ± SD)	78.2 ± 19.1	81.2 ± 8.5	0.04
BMI (mean ± SD)	26.3 ± 7.6	25.3 ± 5.8	0.44
Trough level, mg/L (mean ± SD)	17 ± 7.5	18.9 ± 7.3	0.13
SCr, mg/dL (mean ± SD)	0.69 ± 0.12	0.72 ± 0.13	0.054
Albumin level, g/L (mean ± SD)	28.14 ± 6.2	26.9 ± 5.8	0.25

This table provides a comparative analysis of patients with and without vancomycin-associated nephrotoxicity (VAN), examining demographics and clinical metrics such as gender distribution, age, body mass index (BMI), vancomycin trough levels, serum creatinine (SCr), and albumin levels. Statistical analysis revealed a significant age difference between the groups, while other parameters did not show significant disparities.

## Data Availability

The datasets used and/or analyzed during the present study are available from the corresponding author upon reasonable request.

## Supplementary Materials

The following supporting information can be downloaded at https://www.mdpi.com/article/10.3390/healthcare12111144/s1: References [15,25,26] are cited in the Supplementary Materials.
