# Supplementary material for: Comparing Actual and Rounded Serum Creatinine Concentration for Assessing the Accuracy of Vancomycin Dosing in Elderly Patients: A Single-Center Retrospective Study
Source: Healthcare (Basel). 2024 Jun 4;12(11):1144. doi: 10.3390/healthcare12111144 (PMC11171742; doi:10.3390/healthcare12111144)
Supplement: Supplementary file 1 [file healthcare-12-01144-s001.zip › healthcare-2986474-supplementary.pdf]

Appendex

Vancomycin Initial dosing adjustment in altered renal function and timing of levels				
**CrCl**	**Loading dose**	**Suggested dose**	**Frequency**	**Timing of Trough Levels**
> 90 mL/min<br>1. Age < 35 yr<br>2. Age > 35 yr<br>3. Age 14-18: follow pediatric dosing recommendations	25 to 30 mg/kg	15 mg/kg<br>15 mg/kg	Q8H (Q6H for age <25 + augmented renal function CrCl > 130ml/min)<br>Q12H	Before fifth dose<br>Before fourth dose
50 – 89 mL/min	20 to 25 mg/kg	15 mg/kg	Q12H	Before fourth dose
35-49 mL/min	20 to 25 mg/kg	15 mg/kg	Q24H (Q24H if CrCl closer to 49 ml/min)	Before third dose
21 – 34 mL/min	20 to 25 mg/kg	15 mg/kg	Q24-48H (depending on level)	Before second dose (non-steady state level to avoid supratherapeutic) then

Suggested vancomycin dosing adjustment based on level (goal trough is 10-20mg/dL)	
**If obtained trough level:**	**Suggested Dosing Adjustment**
>20 mg/L (>14 µmol/L)	• Change to Vancomycin dosing to "dose per level."<br>• Obtain another random level in 12-24 hours.<br>• If level is still higher than 20mg/L, use simple proportional method to calculate the new proposed dose (i.e, if trough is 25 and total daily dose is 2000mg, to target a trough of 15m the dose should be 1200mg per day) dose can be rounded to the<br>• nearest increment of 250.
15-20 mg/L (7-14 µmol/L)	No change
<10 mg/L (< 7 µmol/L)	Increase dose and/or increase frequency by using the proportional.

**Definitions**	
Therapeutic vancomycin trough level	A trough level between 10-20 mg/L at steady state, per institutional guidelines
Subtherapeutic trough level	A trough level of less than 10 mg/L
Supratherapeutic trough level	A trough level greater than 20 mg/L
Inappropriate trough time level	Refers to a situation where the timing of the trough level measurement does not align with the recommended guidelines, typically being taken too early which can lead to inaccurate assessment of drug levels.
No trough level	Indicates that a trough level measurement was not taken at all. This lack of data prevents the healthcare team from accurately monitoring and adjusting vancomycin dosing, which is crucial for ensuring therapeutic effectiveness and minimizing toxicity.
Lack of the target trough level	Means that the measured trough level does not fall within the desired therapeutic range (10-20 mg/L). This could indicate that the vancomycin dose is either too high or too low, potentially leading to ineffective treatment or increased risk of toxicity.
Receiving a low dose of vancomycin	Refers to a patient being administered a vancomycin dose that is below the recommended therapeutic level. This underdosing can result in suboptimal treatment outcomes and may not effectively control the infection.
Target maintenance vancomycin dose	A dose that results in a therapeutic trough level at a steady state. This dose was subject to multiple modifications based on the obtained levels until we reach to the therapeutic trough
Rounded SCr	SCr less than 1 mg/dL, rounded to 1 (88.42 mmol/L)
Dosing based on actual SCr	The dose was initially ordered based on the patient's use TBW in mg/kg/dose rounded to the nearest 250 mg increment within the range of 15 to 20 mg/kg/dose, based on the Infectious Diseases Society of America (IDSA). The dose interval was determined according to the patient's CrCl based on actual SCr, per institutional guidelines.
Dosing based on rounded SCr	The dose based on the patient's TBW in mg/kg/dose rounded to the nearest 250 mg increment within the range of 15 to 20 mg/kg/dose. and the CrCl was calculated based on the rounded value. With rounding SCr to 1 mg/dL for patients with a value less than 1, This rounded value is then used to determine the dose interval per institutional guidelines
Unstable renal function	Chronic kidney disease (CKD), end-stage renal disease (ESRD), acute kidney injury (AKI), or documented changes in SCr of 25% before vancomycin dose
Obese patient	BMI greater than 30 kg/m2
Different age groups	Young-old age, from 65 to 74 years old<br>Middle-old age, from 75 to 84 years old

	Old-old age, 85 years old or older[1]
Correlation	The relationship between vancomycin dosing based on the actual, rounded CrCl and TMD was assessed using the Spearman correlation coefficient (r). A higher value of r indicates a stronger correlation between the two variables[2].
Bias	Bias represents the mean difference between the TMD and each of the vancomycin dosing based on the actual, rounded CrCl. A smaller bias indicates that the actual or rounded dose is closer to the TMD on average[2].
Precision	Precision is defined as one standard deviation (SD) of the bias. A smaller value for precision indicates that the differences between vancomycin dosing based on the actual, rounded CrCl and TMD are more consistent[2].
Error	Error is defined as double the standard deviation of the bias divided by the means of the TMD and the equation under study. A smaller error value indicates a better performance of the tested method. An acceptable error between the two methods was defined as 30% or less. If the calculated error is within this threshold, it is considered acceptable[2].
Accuracy	Accuracy was defined as the percentage of vancomycin dosing based on the actual, rounded CrCl within ±10%, ±15%, and ±30% range of the TMD. Higher accuracy percentages indicate that the actual, rounded are closer to the TMD[2].

The creatinine measurement estimate:

Our study, the conventional Jaffe method was employed to measure serum creatinine levels. This method is a well-established colorimetric assay, which operates as follows:

- Chemical Reaction: Creatinine in the blood sample reacts with picric acid under alkaline conditions. This reaction produces a red-colored complex known as creatinine-picrate.
- Measurement: The intensity of the red color, which corresponds to the creatinine-picrate complex, is directly proportional to the creatinine concentration in the sample. This color intensity is quantified using a spectrophotometer.
- Quantification: The spectrophotometer measures how much light of a specified wavelength is absorbed by the sample, providing a numerical value indicative of the creatinine concentration. This value is essential for assessing kidney function, as it reflects the kidneys' ability to filter waste from the blood[3].

References

1. Lee, S.B.; Oh, J.H.; Park, J.H.; Choi, S.P.; Wee, J.H. Differences in Youngest-Old, Middle-Old, and Oldest-Old Patients Who Visit the Emergency Department. *Clin. Exp. Emerg. Med.* **2018**, *5*, 249–255, doi:10.15441/ceem.17.261.
2. Al-Dorzi, H.M.; Alsadhan, A.A.; Almozaini, A.S.; M Alamri, A.; Tamim, H.; Sadat, M.; Al-Swaidan, L.; Elhassan, E.; Arabi, Y.M. The Performance of Equations That Estimate Glomerular Filtration Rate against Measured Urinary Creatinine Clearance in Critically Ill Patients. *Crit. Care Res. Pract.* **2021**, *2021*, 5520653, doi:10.1155/2021/5520653.
3. Toora, B.D.; Rajagopal, G. Measurement of Creatinine by Jaffe's Reaction--Determination of Concentration of Sodium Hydroxide Required for Maximum Color Development in Standard, Urine and Protein Free Filtrate of Serum. *Indian J. Exp. Biol.* **2002**, *40*, 352–354.
